# Effects of the Draft upon the Type of Last Journal

**Published:** 1863-09

**Authors:** 


Effects of the Draft upon the Type of last Journal.—The draft took place in our city at the time of the publication of our last Journal, and though it was destructive and distressing in its results upon individuals and families, yet we have seen nothing which equaled its effects upon the type of our last issue. The conscripts and exempts were about equal in number, and the confusion and riot was truly "Lachrymal."

